# Some Scotch Asylums

**Published:** 1894-01-06

**Authors:** 


### THE INSTITUTIONAL WORKSHOP.

### SOME SCOTCH ASYLUMS.

(BY OUR SPECIAL COMMISSIONER.)

Circumstances have made matters relatively easier for the Lunacy Commissioners in Scotland than they have for the English Board of Lunacy. The comparatively small population in the former country obviates the necessity for large asylums, and so leaves Commissioners and superintendents more freedom and time to develop new methods which may commend themselves for trial. Apart from this the policy of the Scotch Commissioners is, in our view, wiser on certain points than the English system of control. In England the Commissioners insist upon a limit for day-room and dormitory space, they are opposed to the medical superintendent having his private residence distinct from the asylum, and they do not encourage originality on the part of those charged with the treatment of the insane. Hence, it would not be difficult to give an example of a modern asylum, recently erected in England, where the corridors have been made so narrow, owing to the Commissioners inflexible rule as to superficial space that the day-rooms are less efficient, besides being more unattractive than they might and ought to be.

What can it matter to the Board of Lunacy when a County Council chooses to spend a few more pounds in order to give breadth and beauty to the design of their architect, and at the same time to increase the attractiveness of the appearance and consequently the value for treatment of the whole institution? The truth is that there are far too few English Commissioners for the work to be done, the ideas of the present Board need extension and development, and these changes will be best secured by adding to the number of Commissioners, and selecting men of experience and breadth of view for these important duties. The Scotch Board of Lunacy encourage individualism, so far as the medical superintendents are concerned, and in consequence each asylum has its own marked characteristics, and the whole of these institutions are thereby made materially better in all respects than they otherwise would be.

In Scotland, too, the local authorities are encouraged to erect fine buildings, and they would never be prevented from giving adequate space to corridors and day-rooms. Yet the cost per bed of asylum buildings in Scotland is, on the whole, less than it is in England. Again, the Scotch Commissioners hold the view that to enable a man to develop into the highest type of medical superintendent it is essential that his home surroundings shall be distinct from the asylum, and all which appertains to it. No man of experience will question the soundness of the view that those who tend the insane should be afforded the maximum of opportunity, during their hours of leisure, to escape from their surroundings and enjoy the utmost freedom from them. It is a cruelty to insist upon a man of education, in charge of a great lunatic asylum, being so circumstanced as to house accommodation that it is impossible for him to have friends or children in his house without bringing them into contact with the institution and its inmates. If it be contended that the nearer a superintendent is to the asylum the more often he will visit it, the voice of experience replies, "Not at all," for an inefficient officer is made more neglectful still by the compulsory enforcement of conditions which are not only distasteful, but harmful to his own well-being and that of his family. Indeed, from the Commissioners' point of view, within reasonable limits, the further the superintendent's house is from the asylum proper the easier it should surely be for them to ascertain when a particular officer is less attentive to his duties than they may consider necessary. The points we have here dealt with might usefully be made the subject, not only of discussion, but of action on the part of the Council of the Medico-Psychological Association.

### LUNATICS IN WORKHOUSES.

There is a point of difference between the English and Scotch system which we were much interested in examining during a recent visit to Scotland. In Scotland, since 1874, the grant in aid of 4s. a week at present given to Boards of Guardians to pay for pauper lunatics in county asylums, registered hospitals, and licensed houses, has been also given for pauper lunatics (dements and imbeciles) in workhouse wards, provided the accommodation is maintained to the satisfaction of the Commissioners in Lunacy. We paid a visit to the Perth poor-house, in order to inspect the working of this system. We found there forty lunatics, twenty men and twenty women, of the class indicated above. The accommodation was good, the patients were undoubtedly happy and comfortable, and the whole of the arrangements reflected the greatest credit upon the guardians of the poor. We noticed that a good deal of effort had been made to decorate the dormitories, and to make them bright and cheerful in every way. The effect, taken as a whole, was undoubtedly good. We ascertained, however, that the work had been done by a pauper inmate, without his receiving payment for his work, and that the poor-law system did not permit the encouragement of a capable artisan like this, who had fallen into distress, to make an effort to re-establish his position in the world by giving him the means to start afresh, although otherwise able to do so. There is surely something wrong in a system which does not provide for the restoration of the pauper who may be found worthy to a state of independence and respectability outside the workhouse. There can be no doubt that the Commission on the Poor Laws now sitting will be well advised to inquire into this blot, and to recommend the most radical changes in the best interests of all classes of the community.

The system of placing a limited number of harmless cases in the lunatic wards of poor-houses has been attended with the best results in Scotland. Pains are taken to make the wards homelike and comfortable, and all the requirements of the inmates appear to be well supplied. It is the practice to provide out-door work for the men, and some of the women are frequently employed in the laundry. This employment of the patients in suitable and profitable work has many advantages, and helps to promote a high standard of health amongst the inmates. The patients are encouraged to read, and are provided with other amusements, and their accommodation and treatment in these lunatic wards is attended by the most satisfactory results. In such circumstances, having regard to the amount of accommodation available at the present time in many English workhouses, and to the further fact that the accommodation for the insane in asylums in England to-day is inadequate, we are strongly of opinion that the English Board of Lunacy would be well advised to take steps to secure a modification of the present condition of grants in aid in England, so as to enable some of the vacant accommodation in the workhouses to be utilised as lunatic wards. It is noticeable that the Scotch Commissioners strictly limit the number of lunatics admitted to each poorhouse, a fact which we commend to those who may be sufficiently interested to move in favour of securing an alteration in the conditions upon which the grants in aid are at present made in this country.

We cannot in justice close our remarks on this subject without paying a tribute to the wonderful state of order in which we found the Perth poor-house After inspecting the lunatic wards we thought it would. be interesting to visit other parts of the house with the view of testing the accommodation provided. We were glad to find that although in certain respects the lunatic wards were more attractive and possibly more comfortable than the workhouse dormitories, in essential particulars the latter contained every necessary element for comfort. We noted, however, that the lavatory arrangements might be improved, new fittings being requisite, and we formed the impression that the supply of linen, especially towels, was inadequate. The whole establishment was in excellent order, beautifully clean; the inmates seemed happy, comfortable, and well cared for, and on the whole we came to the conclusion that there are worse places to reside in than the Perth poorhouse.

